# Neutrophil-to-Lymphocyte and Platelet-to-Lymphocyte Ratios in Patients with Pterygium: A Retrospective CaseControl Study

**DOI:** 10.3390/jcm15208172

**Published:** 2026-10-21

**Authors:** Ahmet Duhan Özbay, Adem Türk, Gökhan Özgür

**Affiliations:** 1 Department of Ophthalmology, Medicana International Samsun Hospital, Samsun 55080, Türkiye; ahmet.ozbay@medicana.com.tr; 2 Department of Ophthalmology, Faculty of Medicine, Karadeniz Technical University, Trabzon 61080, Türkiye; ademturk@ktu.edu.tr

**Keywords:** pterygium, neutrophil-to-lymphocyte ratio, platelet-to-lymphocyte ratio, systemic inflammation, ocular surface, complete blood count

## Abstract

**Background/Objectives:** Inflammation is implicated in pterygium, but whether routine blood-count indices are associated with pterygium independently of age is unclear. We evaluated the neutrophil-to-lymphocyte ratio (NLR) and platelet-to-lymphocyte ratio (PLR) in systemically healthy patients with pterygium and systemically healthy controls undergoing strabismus surgery. **Methods:** This retrospective case-control study compared preoperative NLR and PLR in 100 patients undergoing pterygium surgery (2010–2019) and 86 systemically healthy adult controls undergoing strabismus surgery. Analyses included Holm-corrected Mann–Whitney tests, age- and sex-adjusted logistic regression, receiver operating characteristic (ROC) analysis, and age-restricted and age-matched sensitivity analyses. **Results:** Patients with pterygium were older (48.3 vs. 29.4 years) and had higher NLR (1.98 vs. 1.66) and PLR (128.1 vs. 114.3) (Holm-adjusted *p* = 0.006 and 0.005, respectively). After adjustment for age and sex, NLR remained associated with pterygium (odds ratio [OR] 3.18 per unit; 95% confidence interval [CI], 1.54–6.57; *p* = 0.002), whereas PLR did not (*p* = 0.106). In 39 age- and sex-matched pairs, differences were directionally similar but not statistically significant. Areas under the ROC curve (AUCs) were 0.632 for NLR and 0.637 for PLR. Neither index differed by pterygium grade or recurrence. **Conclusions:** NLR, but not PLR, was independently associated with pterygium after adjustment for age and sex, although this association was not confirmed in the small matched subsample. Neither index demonstrated sufficient diagnostic performance to serve as a biomarker. Prospective age-matched studies are needed.

## 1. Introduction

Pterygium is a common fibrovascular disorder of the ocular surface characterized by centripetal extension of conjunctival tissue onto the cornea. Ultraviolet (UV) radiation is the most consistently implicated environmental exposure, while oxidative stress, abnormal epithelial and limbal-cell behavior, angiogenesis, extracellular-matrix remodeling, and inflammatory signaling are recognized components of its multifactorial pathogenesis [1,2,3]. Recent reviews continue to emphasize the interaction between UV exposure and molecular pathways involving oxidative stress, growth factors, apoptosis, tumor-suppressor mechanisms, and immune signaling [4].

Inflammation in pterygium is not restricted to the visible lesion. Increased pro-inflammatory mediators and nitric oxide have been demonstrated in tears, serum, and pterygium tissue, and tear cytokine profiles have been associated with clinical features of disease [5,6]. Systemic oxidative stress and genotoxicity have also been reported in patients with pterygium, providing biological plausibility for systemic inflammatory alterations accompanying a predominantly ocular-surface disease [7].

The neutrophil-to-lymphocyte ratio (NLR) and platelet-to-lymphocyte ratio (PLR) are inexpensive indices derived from the routine complete blood count. NLR integrates changes in innate inflammatory activity and adaptive immune-cell representation, whereas PLR additionally reflects platelet participation in inflammatory and tissue-repair pathways. NLR has been extensively studied as a prognostic marker of systemic inflammation in oncologic and other systemic conditions, although its disease specificity is limited [8,9]. In ophthalmology, NLR and/or PLR have been examined in glaucoma, keratoconus, dry eye disease, idiopathic epiretinal membrane, retinal vein occlusion, age-related macular degeneration, and diabetic retinopathy, and systematic reviews and meta-analyses are available for several of these conditions [10,11,12,13,14]. In a meta-analysis of 32 studies, NLR was higher in patients with eye disorders than in controls overall (standardized mean difference 0.53), including a small pooled difference in pterygium (0.14), but not in dry eye disease [11]. In pterygium, however, the published evidence remains heterogeneous. Previous case–control studies have reported higher NLR, higher PLR, or no significant difference in either index [15,16,17]. A 2021 case–control study of age- and sex-matched patients with pterygium and controls similarly found higher NLR in the pterygium group, but no significant difference in PLR or several other hematologic indices [18].

More recent evidence has expanded the question from disease presence to disease severity and recurrence. A 2025 retrospective study of 196 surgically treated patients identified preoperative NLR and pterygium grade as independent factors associated with recurrence [19]. A 2026 study using anterior segment imaging reported associations between systemic immune-inflammation indices and pterygium severity and volume [20]. Conversely, a 2026 age- and sex-matched study comparing primary, recurrent, and healthy groups found no significant differences in NLR or PLR among the groups, highlighting continuing heterogeneity [21]. Most recently, a matched 2026 study reported higher NLR, but not PLR, among patients with recurrent pterygium after conjunctival autograft surgery [22].

These findings are clinically interesting but difficult to interpret because age, environmental exposure, surgical technique, and other systemic factors may influence inflammatory indices. In particular, aging is accompanied by immunosenescence and chronic low-grade inflammation [23,24], and recent evidence indicates that NLR tends to increase with age [25]. Therefore, an observed difference in NLR or PLR between pterygium patients and controls should not be interpreted as disease-specific without addressing age imbalance.

The present study therefore aimed to compare NLR and PLR between systemically healthy patients with pterygium and systemically healthy controls, while explicitly addressing the substantial age difference between groups using multivariable adjustment and age-restricted and age-matched sensitivity analyses. Secondary analyses examined the discriminatory performance of NLR and PLR, their relationship with pterygium grade, and their association with postoperative recurrence. Surgical techniques and the use of antimetabolites were also documented.

## 2. Materials and Methods

### 2.1. Study Design and Ethical Approval

This retrospective case–control study was conducted at the Department of Ophthalmology, Karadeniz Technical University Faculty of Medicine. The study protocol was approved by the Scientific Research Ethics Committee of Karadeniz Technical University Faculty of Medicine (protocol no. 2019/319; decision no. 23; date of approval 18 November 2019; approval letter no. 24237859-882) and was conducted in accordance with the Declaration of Helsinki. Because of the retrospective design and use of anonymized clinical data, the requirement for individual informed consent was waived by the ethics committee. The study was reported in accordance with the Strengthening the Reporting of Observational Studies in Epidemiology (STROBE) recommendations.

### 2.2. Study Population and Eligibility Criteria

Medical records of patients who underwent pterygium surgery between February 2010 and November 2019 were retrospectively reviewed. Patients were eligible if they were systemically healthy, had a complete preoperative ophthalmologic examination and preoperative complete blood count (CBC), and had no ocular comorbidity other than pterygium. Systemic exclusion criteria included diabetes mellitus, hypertension, autoimmune or systemic inflammatory disease, malignancy, hematologic disease, pregnancy, chronic systemic medication use, and other documented chronic systemic conditions. Additional ocular exclusion criteria included ocular or head trauma, previous ocular surgery other than prior pterygium excision in recurrent cases, and clinically significant ocular comorbidity. Patients younger than 20 years were excluded. Of 171 pterygium records screened, 100 patients met the eligibility criteria and were included.

Controls were selected from patients who underwent strabismus surgery at the same institution during the same period. The same systemic eligibility requirements and lower age limit of 20 years were applied. Of 247 candidate records screened, 86 systemically healthy controls were included. The control group was not individually age-matched to the pterygium group; therefore, age was prespecified as an important potential confounder and was incorporated into the multivariable analysis.

### 2.3. Hematologic Parameters and Clinical Variables

Absolute neutrophil, lymphocyte, and platelet counts were obtained from CBC samples collected the day before surgery as part of the routine preoperative evaluation. NLR was calculated as the absolute neutrophil count divided by the absolute lymphocyte count, and PLR as the absolute platelet count divided by the absolute lymphocyte count. The reported NLR and PLR values represent patient-level ratios calculated before group averaging.

Pterygium was classified as primary or recurrent according to previous pterygium surgery. Clinical severity was categorized as grades 2, 3, or 4 in the available records. Postoperative follow-up duration and recurrence were extracted from the medical records. Recurrence was defined according to the clinical diagnosis recorded during postoperative follow-up.

### 2.4. Surgical Technique and Adjuvant Therapy

Surgical technique was recorded for each pterygium case and categorized as bare sclera excision, conjunctival flap, conjunctival autograft, amniotic membrane transplantation (AMT), or combined conjunctival autograft with AMT. The available surgical records did not document the use of antimetabolite adjuvants such as mitomycin C or 5-fluorouracil. All operations were performed by experienced ophthalmic surgeons. Surgeon experience was not quantitatively assessed and was therefore not included as a covariate.

### 2.5. Statistical Analysis

Statistical analyses were performed using IBM SPSS Statistics (IBM Corp., Armonk, NY, USA, Version 31). All statistics were calculated from the patient-level data and verified in Python 3 (Python Software Foundation, Wilmington, DE, USA) using SciPy, statsmodels, and scikit-learn, which were also used for the sensitivity analyses described below. Continuous variables were assessed for distribution. Because the CBC-derived inflammatory variables were not uniformly normally distributed, between-group comparisons were performed using the Mann–Whitney U test. Age was compared using Welch’s independent-samples *t*-test. Sex distribution was compared using the chi-square test. Kruskal–Wallis tests were used to compare NLR and PLR across pterygium grades. Associations of NLR and PLR with postoperative recurrence were explored using Mann–Whitney U tests because only 10 recurrence events were available.

To address the substantial age imbalance between the groups, separate multivariable logistic regression models were constructed with pterygium status as the dependent variable and age, sex, and either NLR or PLR as independent variables. Odds ratios (ORs) are presented with 95% confidence intervals (CIs); they refer to a 1-unit increase in NLR or PLR (and 1 year of age), and standardized ORs per 1 standard deviation (SD) of the cohort distribution are also reported. Because five related hematologic endpoints were compared between groups (neutrophil, lymphocyte, platelet counts, NLR, and PLR), Holm’s step-down procedure was applied to the corresponding Mann–Whitney U-test *p*-values. Receiver operating characteristic (ROC) analysis was performed for NLR and PLR; areas under the ROC curve (AUCs) with 95% CIs were estimated using 10,000 percentile bootstrap resamples. Optimal cutoffs were determined by maximizing the Youden index. All tests were two-sided, and *p* < 0.05 was considered statistically significant unless otherwise specified.

Sensitivity analyses addressed the age imbalance and its modeling. Within-group associations between age and NLR or PLR were assessed with Spearman correlation, and a quadratic age term was added to the logistic models. The contribution of NLR or PLR beyond age and sex was assessed with likelihood-ratio tests, and the apparent AUC of each model’s predicted probability (not cross-validated) was compared with that of age alone and of the age-and-sex model. Comparisons and adjusted models were repeated after restricting both groups to ages ≤45, ≤40, and ≤35 years. Each pterygium patient was also matched 1:1, without replacement, to a control of the same sex and nearest age within 3 years (Mann–Whitney U and Wilcoxon signed-rank tests), and the matching procedure, with random matching order, was repeated in 2000 bootstrap resamples of both groups to evaluate the stability of the result to the selection of controls. For recurrence, each recurrent case was matched to the nearest-age non-recurrent patient(s) of the same sex (1:1 and 1:3). Recurrence rates between the two most frequent surgical techniques and between primary and previously recurrent pterygium were compared with Fisher’s exact test. The comparison of NLR and PLR between recurrent and non-recurrent patients was repeated after excluding patients with less than 12 months of follow-up.

## 3. Results

### 3.1. Demographic Characteristics

The final analysis included 100 patients with pterygium and 86 controls. The mean age was 48.34 ± 12.15 years (range, 20–70) in the pterygium group and 29.36 ± 10.22 years (range, 20–58) in the control group (Welch t = 11.57, *p* < 0.001). There was no significant difference in sex distribution (χ^2^ = 0.46, *p* = 0.497). Among the pterygium patients, 84 (84%) had primary disease and 16 (16%) had recurrent disease at presentation. Pterygium grade was 2 in 43 patients (43%), 3 in 53 (53%), and 4 in 4 (4%) (Table 1).

### 3.2. Complete Blood Count Parameters, NLR, and PLR

**Figure 1:**
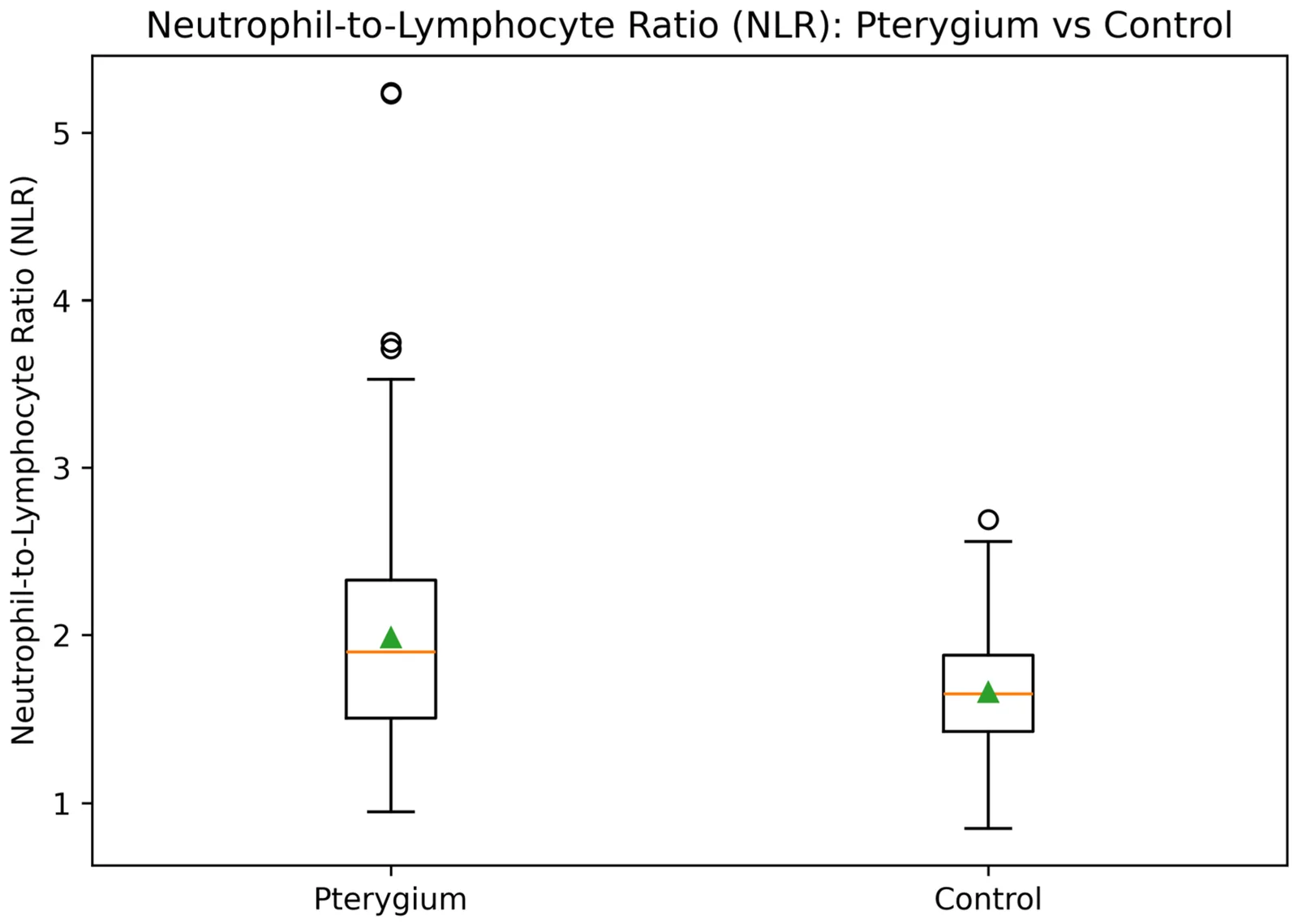
Distribution of NLR by study group. Box plots of NLR in patients with pterygium (*n* = 100) and controls (*n* = 86). The box spans the interquartile range, the horizontal line is the median, the triangle is the mean, whiskers extend to 1.5 times the interquartile range, and circles are outliers. NLR was higher in pterygium (Mann–Whitney U = 5432.0; unadjusted *p* = 0.002, Holm-adjusted *p* = 0.006); the comparison is not adjusted for age.

**Figure 2:**
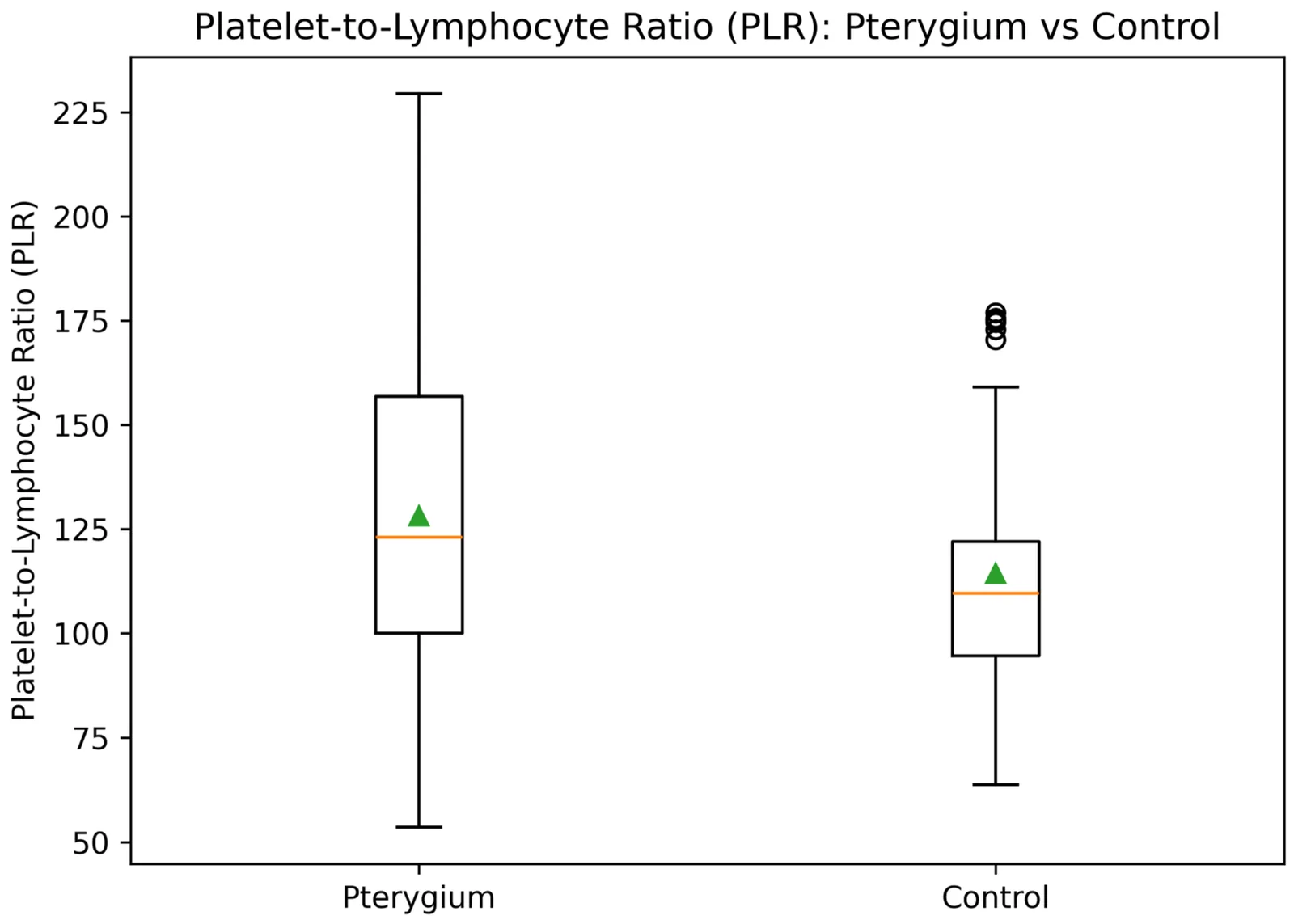
Distribution of PLR by study group. Box plots of PLR in patients with pterygium (*n* = 100) and controls (*n* = 86), with the same conventions as Figure 1. PLR was higher in pterygium (Mann–Whitney U = 5478.0; unadjusted *p* = 0.001, Holm-adjusted *p* = 0.005); this difference did not persist after adjustment for age and sex (Table 3).

Neutrophil counts were higher in the pterygium group than in controls (4.219 ± 1.403 vs. 3.626 ± 0.671 × 10^3^/µL; U = 5784.5, Holm-adjusted *p* < 0.001). Lymphocyte counts did not differ significantly (2.228 ± 0.622 vs. 2.241 ± 0.446 × 10^3^/µL; U = 3830.0, adjusted *p* = 0.200). Platelet counts were modestly higher in the pterygium group (268.56 ± 54.87 vs. 250.28 ± 56.95 × 10^3^/µL; U = 5146.0, adjusted *p* = 0.042). Mean NLR was higher in patients with pterygium than in controls (1.984 ± 0.773 vs. 1.659 ± 0.356; U = 5432.0, unadjusted *p* = 0.002, Holm-adjusted *p* = 0.006) (Table 2). The distribution of NLR by study group is shown in Figure 1. Mean PLR was also higher (128.10 ± 39.13 vs. 114.30 ± 28.26; U = 5478.0, unadjusted *p* = 0.001, Holm-adjusted *p* = 0.005) (Table 2). The distribution of PLR by study group is shown in Figure 2. NLR and PLR values were calculated at the individual-patient level rather than by dividing group-level mean neutrophil or platelet counts by the group-level mean lymphocyte count.

### 3.3. Age- and Sex-Adjusted Associations

Because the pterygium group was substantially older than the controls, separate multivariable logistic regression models were used to assess whether NLR or PLR remained associated with pterygium after adjustment for age and sex. In the NLR model, age (OR 1.156, 95% CI 1.110–1.204, *p* < 0.001) and NLR (OR 3.182, 95% CI 1.540–6.574, *p* = 0.002) were independently associated with pterygium, whereas sex was not (*p* = 0.994). In the PLR model, age remained associated with pterygium (OR 1.146, 95% CI 1.103–1.190, *p* < 0.001), but PLR was not independently associated (OR 1.010, 95% CI 0.998–1.023, *p* = 0.106). Because NLR and PLR are measured on different scales, the odds ratios are also given per 1 SD of the cohort distribution: NLR (SD 0.64), OR 2.09 (95% CI 1.32–3.31); and PLR (SD 35.1), OR 1.44 (95% CI 0.93–2.23). A 1-unit increase in NLR is large relative to its observed distribution, so the per-unit estimate should not be read as the effect of a typical difference (Table 3).

**Table 3:** Multivariable logistic regression models for the association of NLR and PLR with pterygium.

Model/Predictor	OR	95% CI	*p* Value
NLR model: Age	1.156	1.110–1.204	<0.001
NLR model: Sex	0.997	0.436–2.281	0.994
NLR model: NLR	3.182	1.540–6.574	0.002
PLR model: Age	1.146	1.103–1.190	<0.001
PLR model: Sex	0.857	0.386–1.904	0.706
PLR model: PLR	1.010	0.998–1.023	0.106

OR, odds ratio; CI, confidence interval. Separate models were used for NLR and PLR. ORs are per 1-unit increase; per 1 SD of the cohort distribution, NLR OR 2.09 (1.32–3.31) and PLR OR 1.44 (0.93–2.23).

### 3.4. ROC Analysis

**Figure 3:**
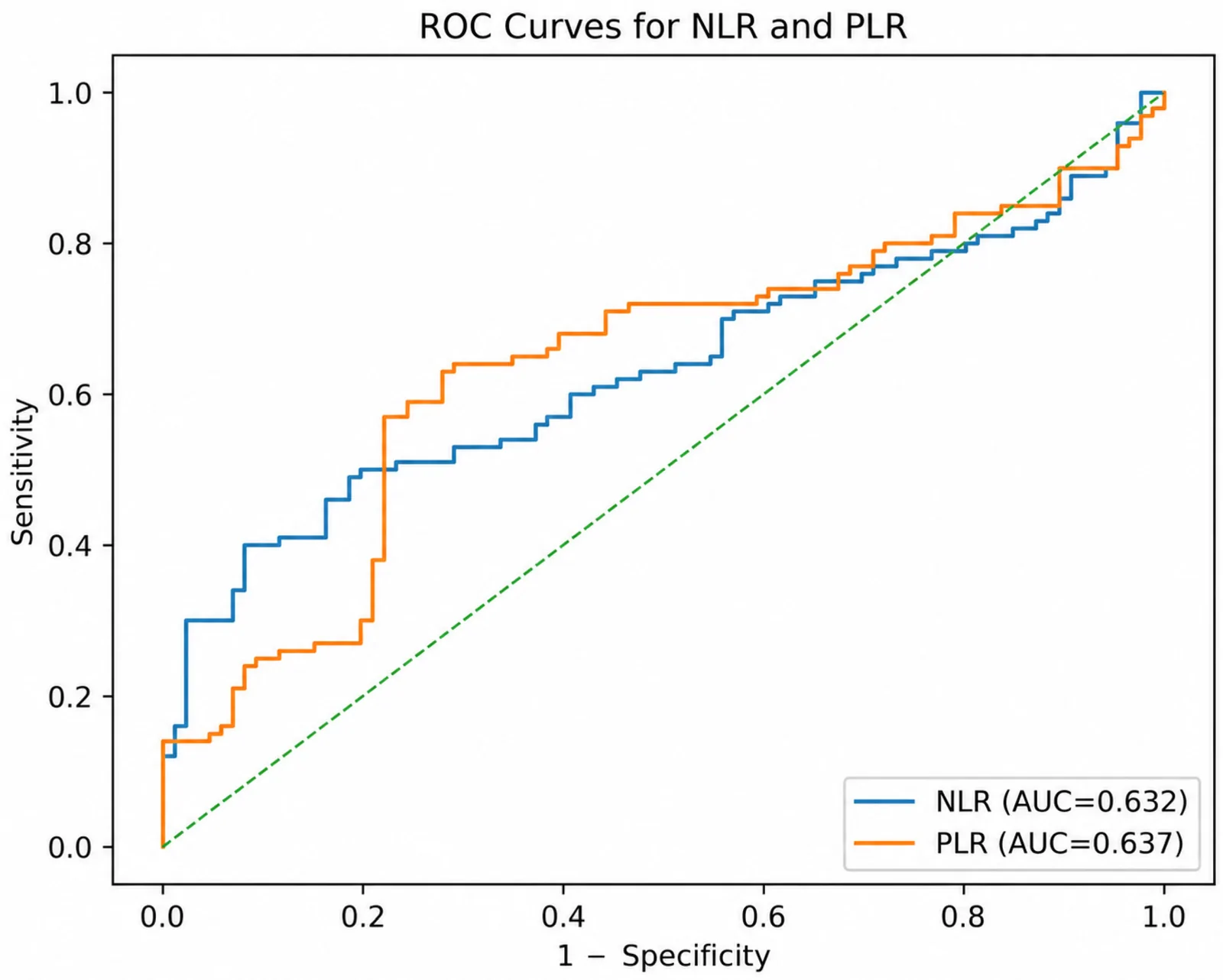
ROC curves for NLR and PLR. Receiver operating characteristic curves for discrimination of pterygium (*n* = 100) from controls (*n* = 86): NLR, AUC 0.632 (95% CI 0.550–0.712); PLR, AUC 0.637 (95% CI 0.556–0.718). The x-axis is 1 − specificity, the y-axis is sensitivity, and the dashed diagonal indicates chance. Youden-optimal cutoffs: NLR 2.107 (sensitivity 40.0%, specificity 91.9%); PLR 119.82 (63.0%, 72.1%). The indices are not adjusted for age; age alone had an AUC of 0.873.

ROC analysis demonstrated modest discrimination between pterygium and control status. The AUC was 0.632 (95% CI 0.550–0.712) for NLR and 0.637 (95% CI 0.556–0.718) for PLR. The Youden-optimal cutoff was 2.107 for NLR, corresponding to 40.0% sensitivity and 91.9% specificity, and 119.82 for PLR, corresponding to 63.0% sensitivity and 72.1% specificity. These values indicate that neither index has sufficient standalone diagnostic accuracy to be considered a diagnostic test (Table 4; Figure 3).

### 3.5. NLR and PLR According to Pterygium Grade

NLR did not differ significantly among grades 2, 3, and 4 (Kruskal–Wallis H = 0.937, *p* = 0.626). Similarly, PLR did not differ significantly by grade (H = 2.538, *p* = 0.281). Grade 4 comprised only four patients; therefore, the absence of a statistically significant difference should not be interpreted as evidence of equivalence across grades (Table 5).

### 3.6. Surgical Technique and Postoperative Recurrence

Mean postoperative follow-up was 43.62 ± 30.82 months (range, 2–109 months). Recurrence occurred in 10 of 100 patients (10.0%); seven had primary pterygium at the index operation and three had recurrent pterygium at presentation. Surgical technique was documented in all 100 pterygium cases (Table 6). The most common techniques were conjunctival flap (64 cases, 64.0%) and conjunctival autograft (29 cases, 29.0%), followed by AMT (4 cases, 4.0%), combined conjunctival autograft plus AMT (2 cases, 2.0%), and bare sclera excision (1 case, 1.0%). The available surgical records did not document the use of antimetabolite adjuvants such as mitomycin C or 5-fluorouracil.

Because several technique categories contained very few patients, formal between-technique recurrence inference was not considered reliable. In the two largest groups, recurrence was observed in 5/64 (7.8%) conjunctival-flap cases and 5/29 (17.2%) conjunctival-autograft cases.

Preoperative NLR did not differ significantly between patients who subsequently developed recurrence and those who did not (1.801 ± 0.762 vs. 2.004 ± 0.775; U = 344.0, *p* = 0.225). PLR was likewise not significantly different (116.57 ± 18.32 vs. 129.38 ± 40.66; U = 392.0, *p* = 0.509). Given only 10 recurrence events, these exploratory analyses were considered underpowered for definitive prognostic inference. In age- and sex-matched comparisons (each recurrent case matched to the nearest-age non-recurrent patient of the same sex), NLR and PLR also did not differ significantly (1:1 matching, *n* = 10 per group: NLR *p* = 0.521, PLR *p* = 0.089; 1:3 matching, *n* = 30 controls: NLR *p* = 0.281, PLR *p* = 0.241). Recurrent and non-recurrent patients were similar in age (47.5 ± 14.7 vs. 48.4 ± 11.9 years, *p* = 0.704). Recurrence did not differ significantly between conjunctival flap and conjunctival autograft (7.8% vs. 17.2%; Fisher’s exact *p* = 0.276) or between primary and previously recurrent pterygium at presentation (7/84 [8.3%] vs. 3/16 [18.8%]; *p* = 0.198); these comparisons are underpowered and descriptive. Restricting the comparison of recurrent and non-recurrent patients to those with at least 12 months of follow-up (*n* = 88, including all 10 recurrences) gave similar results (NLR 1.801 vs. 2.043, *p* = 0.178; PLR 116.57 vs. 130.99, *p* = 0.396).

### 3.7. Sensitivity Analyses for Age Imbalance

Age was not correlated with NLR or PLR within the pterygium group (Spearman ρ = −0.06, *p* = 0.535 for NLR; ρ = 0.00, *p* = 0.995 for PLR), and correlations in controls were weak and not significant (NLR ρ = 0.20, *p* = 0.068; PLR ρ = 0.11, *p* = 0.313). In the pooled cohort, both correlations were weak but significant (NLR ρ = 0.16, *p* = 0.027; PLR ρ = 0.17, *p* = 0.024), as expected from the group age difference. Adding a quadratic age term did not change the NLR estimate (OR 3.17, 95% CI 1.54–6.54; *p* for age^2^ = 0.84) or the PLR estimate (OR 1.010, 95% CI 0.998–1.023; *p* for age^2^ = 0.76). Adding NLR to a model containing age and sex improved model fit (likelihood-ratio χ^2^ = 11.73, df = 1, *p* < 0.001), whereas adding PLR did not (χ^2^ = 2.64, *p* = 0.104). Age alone discriminated pterygium from control with an AUC of 0.873; the apparent AUC of the age-and-sex model was 0.875, and rose to 0.893 with NLR and 0.882 with PLR (not cross-validated).

Restricting the analysis to the age range present in both groups (Table 7), NLR remained significantly higher in pterygium among participants aged ≤45 years (43 pterygium, 79 controls; unadjusted *p* < 0.001; adjusted OR 3.91 (1.61–9.51)). In the narrower strata of ≤40 and ≤35 years, which contained only 24 and 14 pterygium patients, the unadjusted NLR differences were not statistically significant (*p* = 0.201 and 0.074), although the age-adjusted odds ratios were of similar magnitude (3.55 and 3.48). PLR was not associated with pterygium after adjustment in any stratum. In the 1:1 matched sample (39 pairs; mean age 39.9 ± 9.6 vs. 38.2 ± 8.7 years; 17 women in each group) the direction of the differences was unchanged (NLR 2.117 ± 1.000 vs. 1.739 ± 0.417; PLR 125.0 ± 37.9 vs. 117.3 ± 28.3), but they were not statistically significant (Mann–Whitney *p* = 0.187 for NLR and 0.129 for PLR; paired Wilcoxon *p* = 0.066 and 0.309). Across the 1999 usable bootstrap resamples (of 2000) with random matching (median 35 pairs), the median NLR difference was 0.35 (95% interval 0.05 to 0.68), with *p* < 0.05 in 37% of resamples; the corresponding PLR difference was 8.0 (−7.0 to 22.8), with *p* < 0.05 in 41%.

## 4. Discussion

The principal finding of this analysis is that NLR was higher in systemically healthy patients with pterygium than in systemically healthy controls and remained independently associated with pterygium after adjustment for age and sex. PLR was also higher in the unadjusted comparison, but this association was attenuated after age and sex adjustment. This distinction is important because the groups were not age matched and aging itself is associated with chronic low-grade inflammation [23,24] and higher NLR [25]. The NLR association was, however, not statistically significant in an age- and sex-matched subsample of 39 pairs, although the direction and approximate magnitude of the difference were preserved. Our results therefore support an association between NLR and pterygium that should be regarded as hypothesis-generating, and they do not justify interpreting NLR or PLR as disease-specific diagnostic biomarkers.

Our findings are broadly consistent with the existing literature, although the evidence remains heterogeneous. Previous studies have reported higher NLR, higher PLR, or no significant difference in either index [15,16,17,18]. Notably, the age- and sex-matched study by Belviranli et al. also found higher NLR but not PLR [18]. Taken together, these findings suggest that NLR may show a more consistent association with pterygium than PLR, although the influence of age and other population-level differences remains difficult to exclude.

Similar associations have been investigated in other ocular and systemic diseases. A meta-analysis found higher NLR in glaucoma, keratoconus, and idiopathic epiretinal membrane, whereas no significant difference was observed in dry eye disease; the pooled effect for pterygium (standardized mean difference 0.14) was among the smallest significant effects [11]. NLR and PLR have also been studied in age-related macular degeneration, retinal vein occlusion, diabetic retinopathy, and other systemic inflammatory conditions [8,9,10,12,13,14]. This broad use highlights an important limitation of these indices: they are nonspecific, vary with age and comorbidity, and cannot by themselves identify the underlying disorder. A modest association with pterygium is therefore biologically plausible, but should be interpreted as part of a general inflammatory milieu rather than as a pterygium-specific signal.

Several recent studies reinforce the biological plausibility of a relationship between systemic inflammatory indices and pterygium. Chen et al. reported that preoperative NLR and pterygium grade were independently associated with recurrence in 196 surgically treated patients [19]. Niu et al. reported associations between NLR/SII and pterygium severity and volume assessed using anterior segment imaging [20]. In contrast, Alimoğlu et al. found no significant differences in NLR or PLR among primary pterygium, recurrent pterygium, and age- and sex-matched healthy controls [21]. A 2026 matched case–control study of recurrent pterygium after conjunctival autograft surgery reported higher NLR but not PLR in recurrent cases [22]. Taken together, these heterogeneous findings suggest that NLR and related inflammatory indices may be associated with pterygium and its clinical phenotype, but their magnitude and clinical meaning are likely influenced by age, disease phenotype, surgical factors, ocular-surface status, and systemic factors.

Our exploratory grade analysis did not show significant differences in NLR or PLR across grades 2–4. This finding should be interpreted in the context of recent studies suggesting associations between inflammatory indices and pterygium severity or recurrence. Chen et al. reported that pterygium grade was independently associated with recurrence [19], while the 2026 imaging-based study found associations between systemic immune-inflammatory indices and pterygium severity [20]. The present grade analysis should be interpreted cautiously because only four patients had grade 4 disease, and our grading and outcome assessment were based on retrospective clinical records rather than quantitative imaging-derived measurements of pterygium volume. The absence of a significant grade association in our dataset therefore does not exclude a relationship between systemic inflammation and more detailed measures of pterygium burden.

The age imbalance between our groups is a major methodological consideration. The pterygium group was almost 19 years older on average than the control group, and age alone discriminated the groups far better (AUC 0.87) than either inflammatory index. The contemporary literature indicates that aging is accompanied by immunosenescence and inflammaging [23], while systemic inflammation is itself an age-related phenomenon [24], and a 2025 review specifically describes a progressive increase in NLR with age [25]. Age is therefore a plausible alternative explanation for the observed difference. We addressed this through age- and sex-adjusted logistic regression and several sensitivity analyses. Within the pterygium group, age was not correlated with NLR or PLR, and adding a quadratic age term did not materially alter the estimates. In participants aged ≤45 years, NLR remained significantly associated with pterygium, whereas in narrower age strata and in the 1:1 matched sample, the difference remained directionally similar but did not reach statistical significance. In the ≤40 and ≤35-year strata, the unadjusted NLR differences were not significant (*p* = 0.201 and 0.074), whereas the age-adjusted associations remained significant (*p* = 0.009 and 0.020). However, age still differed between groups within these strata, and these analyses included only 24 and 14 pterygium patients, respectively. The adjusted odds ratios therefore should not be interpreted as independent confirmation of the association, particularly because logistic regression estimates are not directly comparable with unadjusted rank-based tests and the restricted samples were small. Similarly, in the 1:1 matched analysis, the NLR difference retained its direction but did not reach statistical significance. In the bootstrap matching analysis, the median NLR difference was 0.35 (95% interval 0.05–0.68), but *p* < 0.05 was observed in only 37% of usable resamples, indicating limited power in matched samples of this size. Taken together, these findings suggest that the NLR association is plausible but sensitive to the method of controlling for age and should therefore be regarded as hypothesis-generating rather than definitive. Statistical adjustment cannot fully reproduce the balance achieved by prospective age-matched recruitment, particularly if the relationship between age and inflammatory indices is nonlinear or affected by unmeasured health and lifestyle factors. Prospective, adequately powered studies with age-matched controls and prespecified confounders are the appropriate next step.

The clinical meaning of the ROC findings also deserves emphasis. The AUCs of 0.632 for NLR and 0.637 for PLR indicate only modest discrimination. Although the optimal NLR cutoff provided high specificity, sensitivity was low; PLR provided more balanced but still limited discrimination. Adding NLR to a model containing age and sex improved model fit (likelihood-ratio *p* < 0.001) but increased the apparent AUC only from 0.875 to 0.893, whereas PLR did not improve model fit (*p* = 0.104). These results argue against using either marker as a standalone diagnostic test. This interpretation is consistent with broader literature emphasizing that NLR and PLR are accessible but nonspecific indicators of systemic inflammatory status [8,9,10].

Regarding clinical relevance, our data do not support using NLR or PLR to diagnose pterygium, to choose between surgical techniques, or to predict recurrence: discrimination was modest, neither index was associated with grade or recurrence, and the design was retrospective. At most, the adjusted association with NLR suggests that systemic inflammatory activity may be associated with pterygium. Whether such an association precedes lesion development or progression, which would be relevant to prevention, cannot be addressed by a case–control design. With respect to treatment and recurrence, the available evidence is heterogeneous [19,21,22], and our exploratory analysis of only 10 recurrence events was underpowered. If NLR proves useful, it would most plausibly enter multivariable recurrence-risk models alongside age, lesion grade and morphology, surgical technique, and ocular-surface parameters rather than act as a stand-alone marker. Because NLR and PLR are inexpensive and already available from preoperative blood counts, prospective multicenter cohorts that also record these variables and local ocular-surface inflammatory markers would be an efficient way to test this. We do not recommend any change in clinical management on the basis of NLR or PLR.

Surgical technique is another important source of heterogeneity in recurrence studies. Our dataset showed that most operations used a conjunctival flap or conjunctival autograft, with smaller numbers receiving AMT, combined autograft plus AMT, or bare sclera excision. The available surgical records did not document the use of antimetabolite adjuvants such as mitomycin C or 5-fluorouracil, and all operations were performed by experienced ophthalmic surgeons. The recent surgical literature continues to support substantial differences in recurrence according to the reconstruction technique, and a 2023 comprehensive review found lower recurrence rates with conjunctival/limbal autografts than with amniotic membrane grafts overall, while combined approaches may perform favorably in selected settings [26]. A 2025 ten-year clinical series likewise reported conjunctival autograft as the predominant technique and emphasized its low recurrence rate [27]. Because several of our technique categories were very small, however, the present study should not be interpreted as a comparative surgical-outcomes study.

Our 10% overall postoperative recurrence rate occurred over a mean follow-up of more than 3.5 years. Baseline NLR and PLR did not differ significantly between recurrent and non-recurrent cases in our cohort, including after age- and sex-matched comparison (all *p* ≥ 0.089). This finding contrasts with the positive association between NLR and recurrence reported by Chen et al. [19] and the 2026 matched study by Kocamış et al. [22]. Differences in surgical technique, recurrence definition, follow-up duration, tear-film status, and case selection may explain part of this heterogeneity. Importantly, our recurrence analysis included only 10 events and therefore has limited statistical power. It should be regarded as exploratory rather than as evidence against a possible prognostic role for NLR.

Environmental exposure is another important limitation. UV radiation remains the most established risk factor for pterygium, and outdoor occupation and cumulative solar exposure are closely related to disease occurrence [28]. Because our retrospective records did not contain standardized measures of cumulative UV exposure, outdoor work, smoking, or other lifestyle factors, residual confounding is possible. The same limitation applies to inflammatory conditions that may have been clinically silent or incompletely documented. We therefore deliberately use the term ‘association’ rather than causal language throughout the manuscript.

The study has additional limitations. Its retrospective single-center design limits generalizability and prevents control of unmeasured confounders. The control group consisted of patients undergoing strabismus surgery rather than community-based healthy volunteers, which may introduce selection effects despite the use of strict systemic eligibility criteria. Because patients younger than 20 years were excluded, the controls are also a selected sample of adult strabismus surgery patients (mean age 29 years) who may differ from the general adult population, and their age distribution is itself part of the imbalance with the pterygium group; the findings may therefore not generalize to older adults without pterygium. The age-matched subsample was small (39 pairs), which limits the power of the matched comparison. The study period spanned ten years, during which clinical practices and laboratory workflows may have changed. Follow-up was heterogeneous (range, 2–109 months; 12 of 100 patients were followed for less than 12 months and 30 for less than 24 months) and no minimum follow-up was required, so late recurrences may have been missed and the recurrence rate may be underestimated. Recurrence was defined as the clinical diagnosis recorded during follow-up, without a prespecified criterion such as a minimum extent (in millimeters) of fibrovascular regrowth onto the cornea, so misclassification of recurrence is possible. The choice of surgical technique was not randomized, and conjunctival flap predominated. Although the available surgical records did not document the use of antimetabolite adjuvants such as mitomycin C or 5-fluorouracil, their use could not be independently verified retrospectively and therefore cannot be completely excluded as a potential source of confounding in recurrence analyses. Although all operations were performed by experienced ophthalmic surgeons, surgeon experience was not quantitatively assessed retrospectively. We also lacked C-reactive protein, cytokine measurements, tear-film inflammatory markers, and quantitative imaging-derived pterygium volume. Finally, the number of grade 4 cases and postoperative recurrences was small. These limitations support the need for prospective multicenter studies with age-matched controls, standardized environmental exposure assessment, objective pterygium imaging, and longitudinal postoperative inflammatory measurements.

Despite these limitations, the study has several strengths. It includes a relatively large single-center pterygium cohort with strict systemic exclusion criteria, a decade-long observation window, patient-level NLR and PLR calculations, explicit adjustment for age and sex together with age-restricted and age-matched sensitivity analyses, ROC analysis, detailed documentation of surgical technique, and long postoperative follow-up. The analysis also places the results within the context of both positive and negative recent studies rather than treating NLR or PLR as established biomarkers.

## 5. Conclusions

In this retrospective case–control study, NLR was higher in systemically healthy patients with pterygium and remained independently associated with pterygium after adjustment for age and sex, whereas the unadjusted PLR difference was attenuated after adjustment. The NLR difference retained its direction but was not statistically significant in a small age- and sex-matched subsample, so the association requires confirmation. Neither NLR nor PLR demonstrated strong standalone discriminatory performance, and neither was significantly associated with pterygium grade or postoperative recurrence in this dataset. These findings suggest a possible association between systemic inflammatory activity and pterygium but should not be interpreted as establishing NLR or PLR as diagnostic or prognostic biomarkers. Prospective, age-matched, multicenter studies incorporating environmental exposure, ocular-surface inflammation, and quantitative pterygium severity measures are needed.

## Figures and Tables

**Table 1:** Demographic characteristics of the pterygium and control groups.

Variable	Pterygium (*n* = 100)	Control (*n* = 86)	Test Statistic	*p* Value
Age, years	48.34 ± 12.15 (20–70)	29.36 ± 10.22 (20–58)	Welch t = 11.57	<0.001
Female, *n* (%)	48 (48.0)	37 (43.0)	χ^2^ = 0.46	0.497
Male, *n* (%)	52 (52.0)	49 (57.0)		
Primary pterygium, *n* (%)	84 (84.0)	—	—	—
Recurrent at presentation, *n* (%)	16 (16.0)	—	—	—
Grade 2, *n* (%)	43 (43.0)	—	—	—
Grade 3, *n* (%)	53 (53.0)	—	—	—
Grade 4, *n* (%)	4 (4.0)	—	—	—

Values are presented as mean ± SD (range) for age and *n* (%) for categorical variables. SD, standard deviation. Age was compared between groups using Welch’s *t*-test, and sex distribution was compared using the chi-square test (one test across the female and male rows). Pterygium grades 2–4 are presented as recorded in the clinical records.

**Table 2:** Complete blood count parameters, NLR and PLR, in the pterygium and control groups.

Parameter	Pterygium (*n* = 100)	Control (*n* = 86)	U Statistic	Raw *p*	Holm-Adjusted *p*
Neutrophils, ×10^3^/µL	4.219 ± 1.403 (2.000–9.120)	3.626 ± 0.671 (2.250–4.940)	5784.5	<0.001	<0.001
Lymphocytes, ×10^3^/µL	2.228 ± 0.622 (1.320–4.310)	2.241 ± 0.446 (1.300–3.480)	3830.0	0.200	0.200
Platelets, ×10^3^/µL	268.56 ± 54.87 (163–412)	250.28 ± 56.95 (150–375)	5146.0	0.021	0.042
NLR	1.984 ± 0.773 (0.95–5.24)	1.659 ± 0.356 (0.85–2.69)	5432.0	0.002	0.006
PLR	128.10 ± 39.13 (53.62–229.49)	114.30 ± 28.26 (63.75–176.89)	5478.0	0.001	0.005

Values are mean ± SD (range). NLR, neutrophil-to-lymphocyte ratio; PLR, platelet-to-lymphocyte ratio. Holm correction was applied across the five between-group hematologic comparisons.

**Table 4:** Receiver operating characteristic analysis of NLR and PLR for discrimination of pterygium from controls.

Marker	AUC	95% CI	Optimal Cutoff	Sensitivity	Specificity
NLR	0.632	0.550–0.712	2.107	40.0%	91.9%
PLR	0.637	0.556–0.718	119.82	63.0%	72.1%

AUC confidence intervals were estimated by 10,000 percentile bootstrap resamples. Cutoffs were selected by the maximum Youden index.

**Table 5:** NLR and PLR according to pterygium grade.

Grade	*n*	NLR, Mean ± SD	PLR, Mean ± SD
2	43	1.974 ± 0.802	124.28 ± 40.38
3	53	1.978 ± 0.777	128.45 ± 36.73
4	4	2.165 ± 0.420	164.54 ± 48.46
Overall test	—	H = 0.937; *p* = 0.626	H = 2.538; *p* = 0.281

Values are presented as mean ± SD. Comparisons across pterygium grades were performed using the Kruskal–Wallis test; H denotes the Kruskal–Wallis test statistic. NLR, neutrophil-to-lymphocyte ratio; PLR, platelet-to-lymphocyte ratio; SD, standard deviation.

**Table 6:** Surgical technique and postoperative recurrence.

Surgical Technique	*n*	Recurrence, *n* (%)
Bare sclera excision	1	0 (0.0%)
Conjunctival flap	64	5 (7.8%)
Conjunctival autograft	29	5 (17.2%)
Amniotic membrane transplantation	4	0 (0.0%)
Conjunctival autograft + AMT	2	0 (0.0%)
Total	100	10 (10.0%)

Values are presented as *n* (%). Recurrence rates between conjunctival flap and conjunctival autograft groups were compared using Fisher’s exact test. The remaining surgical technique groups had no observed recurrences and were not included in the inferential comparison.

**Table 7:** Sensitivity analyses of the NLR and PLR differences between pterygium and control groups.

Analysis	Index	*n* (Pterygium/Control)	Mean, Pterygium vs. Control	Unadjusted *p*	Adjusted OR (95% CI); *p*
Full cohort	NLR	100/86	1.984 vs. 1.659	0.002	3.18 (1.54–6.57); *p* = 0.002
	PLR		128.10 vs. 114.30	0.001	1.010 (0.998–1.023); *p* = 0.106
Age ≤ 45 years	NLR	43/79	2.096 vs. 1.647	<0.001	3.91 (1.61–9.51); *p* = 0.003
	PLR		124.93 vs. 114.41	0.009	1.006 (0.991–1.022); *p* = 0.417
Age ≤ 40 years	NLR	24/70	2.065 vs. 1.647	0.201	3.55 (1.38–9.14); *p* = 0.009
	PLR		117.88 vs. 114.43	0.524	1.003 (0.986–1.020); *p* = 0.766
Age ≤ 35 years	NLR	14/61	2.275 vs. 1.655	0.074	3.48 (1.21–9.97); *p* = 0.020
	PLR		121.31 vs. 114.89	0.484	1.001 (0.981–1.022); *p* = 0.909
1:1 matched (sex exact, age ± 3 y)	NLR	39/39	2.117 vs. 1.739	0.187 (paired 0.066)	—
	PLR		125.00 vs. 117.25	0.129 (paired 0.309)	—

Means are shown for pterygium vs. control. Unadjusted *p*, Mann–Whitney U test. Adjusted OR, per 1-unit increase in NLR or PLR from separate logistic models including age and sex. Matched analysis: each pterygium patient matched 1:1 without replacement to a control of the same sex and nearest age within 3 years; paired *p*, Wilcoxon signed-rank test. Strata ≤ 40 and ≤35 years contained only 24 and 14 pterygium patients.

## Data Availability

The data presented in this study are available from the corresponding author on reasonable request, subject to institutional and ethical restrictions. The dataset is not publicly available because it contains clinical information covered by privacy and ethical requirements.
